# Adipose Insulin Resistance and Circulating Betatrophin Levels in Women with PCOS

**DOI:** 10.1155/2020/1253164

**Published:** 2020-01-21

**Authors:** Yirui He, Wenjin Hu, Gangyi Yang, Huilin Guo, Hua Liu, Ling Li

**Affiliations:** ^1^Key Laboratory of Diagnostic Medicine (Ministry of Education) and Department of Clinical Biochemistry, College of Laboratory Medicine, Chongqing Medical University, Chongqing 400016, China; ^2^Department of Endocrinology, The Second Affiliated Hospital, Chongqing Medical University, Chongqing 400016, China; ^3^Chongqing Prevention and Treatment Hospital for Occupational Diseases, Chongqing 400000, China; ^4^Department of Pediatrics, University of Mississippi Medical Center, 2500 North State Street, Jackson, MS, USA

## Abstract

The role of IR and metabolic disorders has become a crucial topic of study in the pathogenesis of PCOS. Adipose tissue is an important target organ of insulin, and adipose IR plays an important role in the occurrence and development of PCOS. This study seeks to investigate the role of adipose IR in the development of PCOS and to examine its relationship with circulating betatrophin levels in women with PCOS. A cross-sectional analysis of a cohort of women with PCOS and healthy women was performed in this study. Serum betatrophin concentrations were measured by ELISA. Adipose IR was calculated using the product of fasting insulin and FFA concentrations, and the relationship between adipose IR, circulating betatrophin, and other parameters was analyzed. Adipose IR in women with PCOS was significantly higher than that in controls. We found that women with PCOS who have adipose IR (adipose IR ≥ 55) have a higher BMI and higher blood glucose, insulin, PRL, FFA, TG, HOMA-_IR_, AUC_glucose_, AUC_insulin_, VAI_female_, and BAI levels than PCOS-afflicted women without adipose IR, while *M*-values, and SHBG and LH levels were lower. In women with PCOS, serum betatrophin levels were significantly increased compared with controls. Adipose IR negatively correlated with *M* values and positively with circulating betatrophin levels in the study population. After metformin treatment, circulating betatrophin levels and adipose IR in women with PCOS were significantly decreased compared with pretreatment. Adipose IR is associated with betatrophin levels in women with PCOS. The combination of adipose IR and circulating betatrophin measurements may be significant for screening patients with PCOS.

## 1. Introduction

Polycystic ovary syndrome (PCOS) is the most common endocrine disorder among women and affects about 10% of reproductive-aged women [1]. Hyperandrogenism, menstrual disorders, and polycystic ovaries are three important characteristics of PCOS [2]. The role of insulin resistance (IR) and metabolic disorders in the pathogenesis of PCOS has been gaining more and more attention [3, 4]. More than 50% of women with PCOS also have metabolic syndrome (MetS) and/or obesity [5, 6]. In addition, about 70% of women with PCOS have IR and related metabolic disorders [7], which results in hyperinsulinemia and increases the risk of type 2 diabetes mellitus (T2DM) and cardiovascular disease (CVD) [8, 9]. Furthermore, the incidence of gestational diabetes (GD) in women with PCOS and IR is also elevated compared to healthy women [10]. However, the underlying mechanisms leading to IR and metabolic disorders in women with PCOS remain unknown. Adipose tissue is an important target of insulin signaling *in vivo* [11]. In recent years, the role of IR-related cytokines in the pathogenesis of PCOS has attracted much attention [3, 12]. Therefore, in PCOS patients, the study of adipose tissue IR (adipose IR) is of great significance.

Betatrophin, also known as angiopoietin-like protein (ANGPTL), is highly conserved in all mammalian species [13]. This protein is mainly expressed in the liver and adipose tissue in rodents [14]. In previous human and animal studies, betatrophin has been found to be related to IR and *β*-cell proliferation [15, 16]. In a previous study, specific depletion of *β*-cells in the pancreas did not cause betatrophin upregulation, suggesting that betatrophin levels might be regulated by IR and not insulin deficiency [17]. Moreover, in insulin-deficient mice and those with type 1 diabetes (T1D), blood betatrophin concentrations were significantly elevated [15, 16]. In mice with obesity and T2DM, mRNA levels of betatrophin in the liver were markedly upregulated [15, 16]. In obese and T2DM human subjects, serum betatrophin levels showed an increase or a decrease [18–21]. Therefore, the current results are inconsistent. In a previous study, we found that insulin stimulation in hepatocytes increased betatrophin expression. Metformin and rosiglitazone treatments led to a reduction in betatrophin expression in insulin-stimulated hepatocytes [12]. These results of the *in vitro* study indicated that betatrophin is regulated by insulin levels.

In the current work, serum betatrophin concentrations are measured, and adipose IR index is calculated in women with PCOS, and the relationship between circulating betatrophin level and adipose IR is analyzed.

## 2. Subjects and Methods

### 2.1. Individuals

A total of 199 individuals participated in the study, including 100 PCOS women and 99 normal women. Patients with PCOS were diagnosed by the Rotterdam criteria, as previously reported [22]. The following diseases were excluded, including late-onset congenital adrenal hyperplasia, 21-hydroxylase deficiency, thyroid dysfunction, Cushing's syndrome, androgen-secreting tumors, and hyperprolactinemia. Ninety-nine healthy women were recruited from the society, secondary schools, or colleges through routine medical examinations or advertisements. These women had a normal menstrual cycle, 21–35 days interval, normal progesterone level in the luteal phase, and no hirsutism, acne, alopecia. The exclusion criteria are as follows: age >40 years, body mass index (BMI) ≥37 kg/m^2^, CVD, thyroid diseases, tumors, smoking, impaired glucose tolerance (IGT), T2DM, hypertension, and kidney diseases. In the past three months of the study, all subjects did not take hormones or drugs that affect insulin sensitivity. And 30 patients with PCOS attended the interventional study of metformin treatment. These patients were given 6 months of oral metformin (850 mg b.i.d.) [3]. Consent was obtained from each patient after full explanation of the purpose and nature of all procedures used. All procedures were performed in compliance with relevant laws and institutional guidelines, and the study was performed in accordance with the Helsinki Declaration and approved by the National Ethics Committee. The privacy rights of human subjects were always observed.

### 2.2. Oral Glucose Tolerance Test (OGTT)

At 8 a.m. on the study day, after a 12-hour overnight fasting, OGTTs were performed in all study populations. These individuals were given 75 grams of oral glucose, and blood samples were taken at designated time points (0, 30, 60, and 120 min) to measure glucose and other biochemical indicators.

### 2.3. Euglycemic-Hyperinsulinemic Clamp (EHC)

We performed EHC in all study population, including 100 PCOS women and 99 normal women, as previously reported [23]. Briefly, ten hours after fasting, a venous catheter was inserted into the antecubital vein for insulin and glucose infusion. Another catheter was inserted into the contralateral dorsal hand vein for blood extraction. Insulin (1 mU/kg/min) was infused for 2 hours. During the EHC, blood glucose was measured every 15 minutes, and 20% glucose was infused to maintain blood glucose at the fasting level. *M* values were calculated by the glucose infusion rate (GIR) and body weight (BW). Blood was withdrawn at indicated time points and stored at −80°C until used.

### 2.4. Anthropometric Measurements

BMI was calculated as weight divided by height squared. Waist-hip ratio (WHR) was calculated by waist circumference (WC) and hip circumference (HC). The homeostasis model assessment of IR (HOMA-_IR_) was calculated based on previously published formulas (HOMA-_IR_ = insulin/(22.5*e*^−ln glucose^)) [24]. AUC_glucose_ and AUC_insulin_ (the area under the curve for glucose and insulin) were accounted following the trapezoidal rule [25]. Body adiposity index (BAI) was calculated as [HC (cm)/(height (m)^1.5^ − 18] [26]. Visceral adiposity index (VAI) = WC/[36.58 + (1.89 × BMI)] × [triglyceride (TG)/0.81] × [1.52/high-density lipoprotein cholesterol (HDL-C)]. Adipose IR was calculated by multiplying the fasting free fatty acid (FFA) concentration (mmol/l) by the fasting insulin concentration (pmol/l) [adipose IR = fasting FFA (mmol/l) × fasting insulin (pmol/l)] [27].

### 2.5. Hormonal and Biochemical Measurement

Blood glucose was measured by the glucose oxidase method. Insulin was measured by the enzyme-linked immunosorbent assay (ELISA). Free fatty acid (FFA), total cholesterol (TC), HDL-C, low-density lipoprotein cholesterol (LDL-C), and TG were analyzed as previously described [3]. Serum luteinizing hormone (LH), follicle-stimulating hormone (FSH), testosterone (TEST), progesterone (Prog), prolactin (PRL), dehydroepiandrosterone sulfate (DHEA-S), and sex hormone-binding globulin (SHBG) were measured as previously described [3, 4]. The free androgen index (FAI) was calculated by [testosterone/sex hormone-binding globulin (SHBG)] ×100.

### 2.6. Betatrophin Measurements

Betatrophin levels were measured with an ELISA kit (Phoenix Pharmaceuticals Inc., Belmont, CA, USA) according to the manufacturer's instruction. The intra-assay and interassay variations were <10% and <15%, respectively. The linear range of the measurement was 0–100 *μ*g/L. The method has high sensitivity and specificity for measuring betatrophin in serum without cross-reaction and interference [28].

### 2.7. Statistical Analysis

All analyses were performed with SPSS version 2.0. All data were expressed as median (interquartile range). The Kolmogorov–Smirnov test was used to examine the distribution of the data. The adipose IR of nonnormal distribution was skewed and logarithmically transformed to obtain a normal distribution. Comparisons between groups were performed by ANOVA, unpaired *t*-test, or paired *t*-test as appropriate. The relationship between parameters was analyzed by Spearman correlation and multiple regression analysis. Binary logistic regression analysis was used to investigate the relationship between adipose IR and PCOS and *M* values. Receiver operating characteristic (ROC) curve of subjects was drawn by using SPSS 2.0 software to evaluate the sensitivity and specificity of adipose IR in the diagnosis of PCOS. The distribution of adipose IR and betatrophin was further divided into four tertiles (quartiles), and odds ratio (OR) was calculated using multivariate logistic regression analysis.

Sample size was calculated using the following equation: *N* = [*Z*_*α*/2_*σ*/*εμ*]^2^ (*σ*, standard; *μ*, mean; *Z*_*α/2*_ = 1.96; *α* = 0.05; and *ε* = 10%). According to the formula, the required sample size is 185, while the actual sample size in the current study is 199. In all statistical tests, *p* < 0.05 was considered as significant.

## 3. Results

### 3.1. Clinical Characteristics, Circulating Betatrophin, and Adipose IR in the Study Individuals

**Figure 1:**
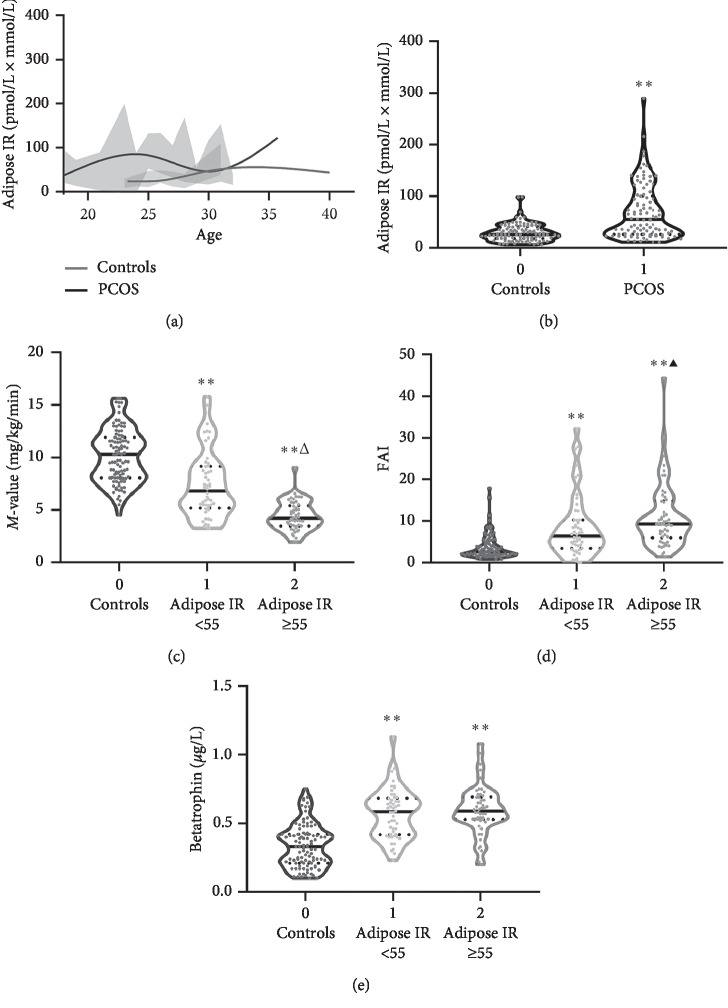
Adipose IR and clinical features in the study population. (a) The curve of adipose IR distribution with age in the study population. (b) Adipose IR levels in normal and PCOS patients. (c) *M* values in normal and PCOS patients with or without adipose IR. (d) FAI in normal and PCOS patients with or without adipose IR. (e) Circulating betatrophin levels in normal and PCOS patients with or without adipose IR. Values were given as median (interquartile range). ^*∗*^*p* < 0.05, ^*∗∗*^*p* < 0.01 compared with controls. ^*▲*^*p* < 0.05,^*△*^*p* < 0.01 compared with PCOS patients without adipose IR.

The anthropometric and laboratory parameters of the individuals are shown in Table 1. In individuals with PCOS, systolic blood pressure (SBP), WHR, fasting blood glucose (FBG), 2 -hour post-glucose load blood glucose (2h-BG), fasting insulin (Fins), 2 h plasma insulin after glucose overload (2h-Ins), FFA, TC, TG, LDL-C, HOMA-_IR_, AUC_glucose_, AUC_insulin_, VAI, and BAI were significantly elevated, whereas *M* values were markedly reduced compared to that in healthy subjects. In addition, in women with PCOS, serum sex hormonal levels, including TEST, LH, PRL, and FAI, were markedly increased, while SHBG was decreased compared with that in healthy women. However, circulating betatrophin was higher in the PCOS patients than in controls. The curve of adipose IR distribution with age in women with PCOS and healthy controls is displayed in Figure 1(a). Importantly, in patients with PCOS, the levels of adipose IR were significantly higher than those in normal women (55.8 (26.0–102.8) vs. 25.7 (18.1–40.0), *p* < 0.01; Figure 1(b)). We divided PCOS women into two subgroups based on adipose IR: *Q*1, adipose IR < 55 and *Q*2, adipose IR ≥ 55, which was defined as having adipose IR. The results showed that PCOS women with adipose IR ≥ 55 had higher BMI, blood glucose, insulin, FFA, TG, HOMA-_IR_, AUC_glucose_, AUC_insulin_, VAI_female_, and BAI than women with PCOS who had adipose IR < 55, while *M* values were lower (Table 1, Figure 1(c)). Furthermore, serum SHBG and LH levels in women with PCOS and adipose IR were lower than those in women with PCOS who had no adipose IR, whereas serum PRL and FAI levels tended to be higher (Table 1, Figure 1(d)). In PCOS patients with or without adipose IR, serum betatrophin concentrations were significantly increased compared with controls (Figure 1(e)).

### 3.2. Correlation between Adipose IR and Other Indicators

In all study women, simple linear correlation analysis of the pooled data showed that adipose IR positively correlated with age, BMI, SBP, WHR, TG, TC, LDL-C, FFA, HOMA-_IR_, AUC_glucose_, AUC_insulin_, VAI_female_, and BAI. Importantly, in our study population, adipose IR was negatively correlated with *M* values and positively correlated with circulating betatrophin levels. In addition, we analyzed the relationship between adipose IR and sex hormone levels and found that adipose IR was positively correlated with DHEA-S, TEST, LH, FAI, and PRL and negatively correlated with SHBG. Multivariate regression analyses showed that circulating betatrophin levels, WHR, *M* values, FFA, AUC_insulin_, BMI, and HOMA-_IR_ were independent factors influencing adipose IR. The regression equation was as follows: *Y*_lg10(adipose IR)_  =  0.577  +  0.784 *X*_FFA_  −  0.012*X*_M_  +  0.287*X*_WHR_  +  0.058*X*_HOMA-IR_  +  0.001*X*_AUC insulin_  +  0.109*X*_Betatrophin_  +  0.006*X*_BMI_.

Binary logistic regression analysis further revealed that adipose IR was significantly related to *M* value and PCOS, even after controlling for anthropometric, metabolic, and hormonal variables and so on (Table 2).

### 3.3. Relationship between Increased Adipose IR and PCOS

**Figure 2:**
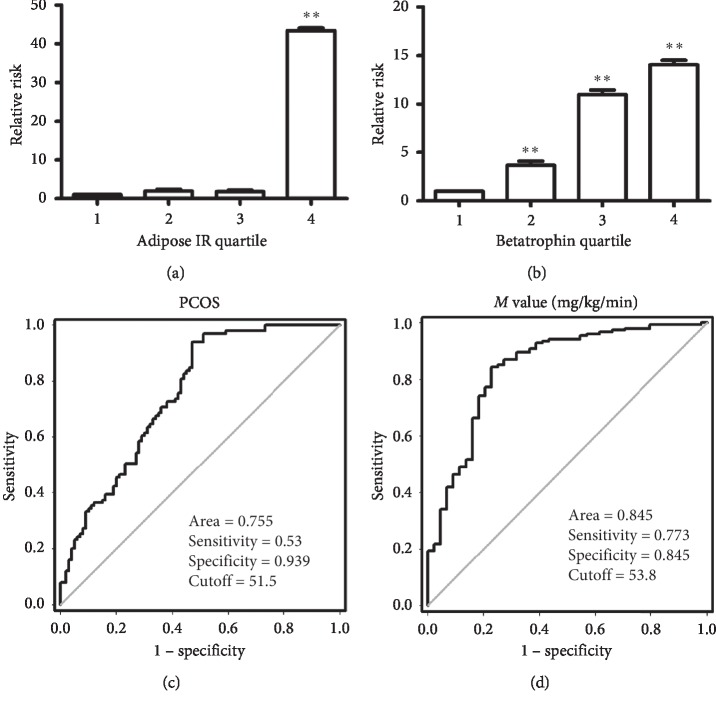
Adipose IR levels and ROC curve analysis in study population (a) Prevalence of elevated PCOS in different quartiles of adipose IR : *Q*1, <21.86; *Q*2, 21.86–33.62; *Q*3, 33.62–63.42; and *Q*4, >63.42 (vs. *Q*1: ^*∗∗*^*p* < 0.01. (b) Prevalence of elevated adipose IR in different quartiles of betatrophin: *Q*1, <0.30 *μ*g/L; *Q*2, 0.30–0.44 *μ*g/L; *Q*3, 0.44–0.62 *μ*g/L; and *Q*4, >0.62 *μ*g/L (vs. *Q*1: ^*∗∗*^*p* < 0.01). (c) ROC curve analyses for the prediction of PCOS according to the adipose IR levels. (d) ROC curve analyses for the prediction of IR (*M* values) according to the adipose IR levels.

We divided adipose IR into four tertiles (*Q*1, <21.86; *Q*2, 21.86–33.62; *Q*3, 33.62–63.42; and *Q*4, >63.42) and calculated the odds of developing PCOS by logistic regression analysis. In tertile 4 of adipose IR, the odds ratio of developing PCOS was the highest (95% CI 11.49; 163.76). When comparing the odds ratio of tertile 4 with tertile 1, the *p* value was *p* < 0.01 (Figure 2(a)).

We also divided betatrophin into four tertiles (*Q*1, <0.30 *μ*g/L; *Q*2, 0.30–0.44 *μ*g/L; *Q*3, 0.44–0.62 *μ*g/L; and *Q*4, >0.62 *μ*g/L). In tertiles 2, 3, and 4 of betatrophin, the odds ratios of developing adipose insulin resistance were gradually increased when compared with tertile 1: (95%CI 1.49, 8.82) for tertile 2, (95%CI 4.51, 26.73) for tertile 3, and (95%CI 5.66, 34.82) for tertile 4 vs. tertile 1, *p* for trend <0.001 (Figure 2(b)).

### 3.4. Receiver Operating Characteristic (ROC) Curve Analysis

To determine the predictive value of adipose IR for PCOS and IR (*M* value), we analyzed the ROC curves of adipose IR. We found that the area under the ROC curves (AUC) was 0.76 (*p* < 0.01), with a sensitivity of 53% and specificity of 94% for PCOS (Figure 2(c)). The AUC was 0.85 (*p* < 0.01) with a sensitivity of 77% and specificity of 85% for IR (*M* values) (Figure 2(d)). The best cutoff values for adipose IR to predict PCOS and IR were 51.5 *µ*g/L and 53.8 *µ*g/L.

### 3.5. Metformin Treatment Improved Adipose IR and Decreased Circulating Betatrophin Levels in PCOS Women

**Figure 3:**
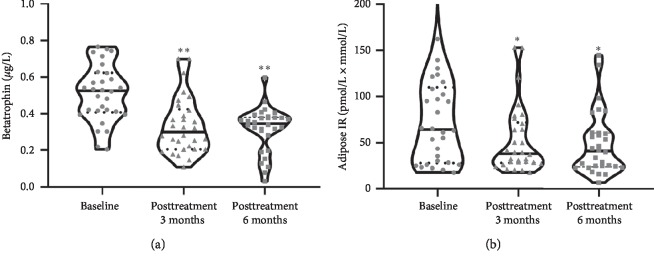
Adipose IR and circulating betatrophin levels in PCOS patients after both 3 and 6 months of metformin treatment. Values were given as median (interquartile range). ^*∗*^*p* < 0.05, ^*∗∗*^*p* < 0.01 vs. baseline.

Table 3 shows the clinical characteristics, laboratory parameters, and adipose IR levels before and after treatment with metformin in PCOS women. We found that BMI, DBP, FBG, 2h-BG, FIns, 2h-Ins, TG, TC, FFA, HOMA-_IR_, AUG_insulin_, VAI_female_, LH, and FAI were significantly decreased, whereas *M* value and SHBG were markedly increased after treatment, as compared with pretreatment (Table 3). After metformin therapy, circulating betatrophin levels decreased markedly in patients with PCOS (Figure 3(a)). Importantly, with the amelioration of whole-body IR, metformin therapy also significantly reduced adipose IR (Figure 3(b)).

## 4. Discussion

IR is an important pathophysiological basis for metabolic disorders in patients with PCOS. In addition to IR in the liver and muscle tissues, IR in fat tissue is also an important feature in PCOS subjects, especially in obese women [29, 30]. In the current study, we found that compared with controls, adipose IR was markedly increased in women with PCOS, and it positively correlated with HOMA-_IR_ and negatively correlated with *M* value, suggesting that adipose IR is an important component of systemic IR, and it may reflect the degree of systemic IR. In addition, adipose IR was found to be positively correlated with TEST and negatively correlated with SHBG. Our results are similar to a previous study where Daniel et al. found that adipose IR was markedly increased in nonobese women with PCOS. The analysis of subcutaneous (SC) abdominal adipocyte size showed an increased percentage in small SC adipocytes in nonobese PCOS women. In addition, they also found that adipose IR positively correlated with HOMA-_IR_ and serum TEST. Therefore, our results confirm that adipose IR is a good marker for screening IR and hyperandrogenism in PCOS-afflicted women. However, some limitations of Daniel et al.'s study should be taken into consideration: (1) a small sample size, including only 10 women with PCOS and 18 controls; (2) only women of normal weight were recruited in that study, without obese women; (3) the EHC, the “gold standard” measure of insulin sensitivity, was not preformed to assess insulin sensitivity in the study population; and (4) the association between adipose IR and cytokines was not studied. However, in the current study, these limitations were eliminated. In addition, the strength of our study is that circulating betatrophin levels were found to be positively associated with adipose IR and independently influenced adipose IR. Importantly, with the increase in circulating betatrophin concentration, adipose IR also increased significantly. These results are consistent with most previous studies, which reported increased serum betatrophin levels in IR individuals or women with PCOS [31–34]. In a previous study, Adamska et al. reported that betatrophin was closely related to IR and islet *β*-cell secretion. Our results further confirm that betatrophin was associated with whole-body IR and adipose tissue IR, as well as insulin levels [35]. Therefore, high betatrophin levels may be a predictor of IR and PCOS. We believe that circulating betatrophin has an important clinical implication for reflecting adipose IR. However, it is not known how adipose IR leads to an increase in circulating betatrophin levels. We speculate that IR may be responsible for the increased compensatory secretion and release of betatrophin in adipose tissue. Furthermore, under the IR conditions, inflammatory cytokines secreted by macrophages may promote the expression and synthesis of betatrophin [12].

Finally, the ROC analyses were performed to investigate the best cutoff point of adipose IR for the detection of *M* values (IR) and the presence of PCOS. Our data revealed that adipose IR might be a good marker for both systemic IR and PCOS. In a previous study, circulating betatrophin has been found to be associated with PCOS [12]. To date, there are no commonly accepted parameters for the screening of PCOS. In addition, the diagnostic criteria for PCOS are also controversial. Therefore, we believe that the combination of adipose IR and circulating betatrophin can serve as an epidemiological tool in screening women for PCOS. Importantly, we determined the optimal cutoff point of adipose IR for screening PCOS. This cutoff point, combined with the cutoff point of circulating betatrophin [12], will increase the accuracy of PCOS screening.

In the intervention study, we found that metformin treatment significantly improved adipose IR and reduced serum betatrophin and FAI levels, further suggesting that adipose IR is related to betatrophin and sexual hormone disorders.

The mechanism by which metformin improves adipose IR and reduces serum betatrophin is not known. We speculate that it is possible that metformin treatment improved IR, resulting in a decrease in betatrophin secretion. However, direct inhibition of betatrophin synthesis and secretion by metformin cannot be excluded. Further investigation is required to address this issue.

Some limitations of the current study include the following: (1) the nature of the cross-sectional study prevents us from mechanistic explanations; (2) the relatively small sample size, which diminished statistical power; (3) the single-ethnicity study population, which limited the ability to apply findings to individuals of different ethnicities; (4) some factors influencing IR, such as physical activity and diet, were not assessed; and (5) finally, the characteristics of adipose tissue in the study population were not compared. Therefore, the current study fails to provide insights into the molecular mechanisms resulting in adipose IR. However, the advantages of this work are as follows: (1) unlike previous reports [11], both obesity and normal weight women participated in this study and (2) all PCOS patients were newly diagnosed; therefore, the influence of confounding factors such as the course of the disease and previous treatments was removed.

In summary, our study shows that individuals with PCOS had significantly increased adipose IR and increased circulating betatrophin levels, and adipose IR was significantly correlated with betatrophin levels, obesity, and FAI. Adipose IR and betatrophin may be two indicators related to hyperandrogenism and systemic IR in women with PCOS. Therefore, this combination can serve as an epidemiological tool for screening PCOS. Due to the increase in the prevalence of PCOS, our data have important public health and clinical implications.

## Figures and Tables

**Table 1:** Main clinical features and laboratory parameters in the study population.

Group	Controls (*n* = 99)	PCOS (*n* = 100)	
		*Q*1 (adipose IR < 55) (*n* = 50)	*Q*2 (adipose IR ≥ 55) (*n* = 50)
Age (yr)	25.0 (24.0–27.0)	27.0 (24.0–30.0)	26.0 (24.0–28.0)
BMI (kg/m^2^)	20.0 (18.6–21.3)	22.5 (19.5–24.9)^*∗∗*^	26.4 (23.4–28.7)^*∗∗*^^∆^
SBP	108.0 (102.0–115.5)	115.5 (108.0–120.0)^*∗∗*^	118.5 (109.0–124.0)^*∗∗*^
DBP	76.0 (70.0–80.0)	75.0 (72.0–80.0)	78.0 (72.0–82.0)
WHR	0.78 (0.75–0.84)	0.85 (0.79–0.89)^*∗∗*^	0.86 (0.82–0.91)^*∗∗*^
*M* value (mg/kg/min)	10.3 (8.1–11.8)	6.8 (5.2–9.1)^*∗∗*^	4.2 (3.5–5.4)^*∗∗*^^∆^
FBG (mmol/L)	4.42 (4.03–4.71)	4.80 (4.33–5.08)^*∗∗*^	5.05 (4.73–5.62)^*∗∗*^^∆^
2h-BG (mmol/L)	5.25 (4.59–6.20)	6.12 (5.23–7.29)^*∗∗*^	8.01 (6.78–10.03)^*∗∗*^^∆^
FIns (mU/L)	7.0 (6.1–8.5)	9.0 (6.5–10.5)	20.9 (17.8–27.2)^*∗∗*^^∆^
2h-Ins (mU/L)	35.7 (22.4–57.0)	81.5 (58.2–113.3)^*∗∗*^	149.2 (126.1–215.2)^*∗∗*^^∆^
FFA (umol/L)	0.52 (0.35–0.75)	0.48 (0.37–0.58)^*∗*^	0.72 (0.57–0.79)^*∗∗*^^∆^
TC (mmol/L)	3.74 (3.15–4.42)	4.40 (3.67–5.21)^*∗∗*^	4.77 (4.16–5.10)^*∗∗*^
TG (mmol/L)	0.79 (0.58–1.27)	1.06 (0.82–1.69)	1.66 (1.10–2.30)^*∗∗*^^▲^
HDL-C (mmol/L)	1.16 (0.97–1.42)	1.27 (1.14–1.54)^*∗*^	1.23 (1.07–1.40)
LDL-C (mmol/L)	2.15 (1.58–2.58)	2.60 (1.97–3.11)^*∗*^	2.73 (1.99–3.19)^*∗∗*^
HOMA-_IR_	1.35 (1.16–1.73)	1.76 (1.45–2.37)	4.83 (4.04–5.99)^*∗∗*^^∆^
AUC_glucose_	11.7 (10.6–13.4)	13.3 (12.4–16.0)^*∗∗*^	16.57 (14.45–19.16)^*∗∗*^^∆^
AUC_insulin_	107.5 (62.6–144.5)	152.8 (131.6–187.1)	262.3 (215.9–352.5)^*∗∗*^^∆^
VAI_female_	1.27 (0.87–2.10)	1.52 (0.99–2.37)	2.35 (1.56–3.63)^*∗∗*^^∆^
BAI	26.8 (24.4–28.1)	27.4 (25.0–30.5)	31.3 (28.3–34.3)^*∗∗*^^∆^
Betatrophin (*μ*g/L)	0.33 (0.21–0.42)	0.59 (0.42–0.68)^*∗∗*^	0.59 (0.53–0.69)^*∗∗*^
SHBG (nmol/L)	57.7 (42.1–75.0)	36.3 (23.7–59.8)^*∗∗*^	30.2 (20.0–40.6)^*∗∗*^^∆^
DHEA-S (*μ*g/dl)	181.3 (141.2–216.3)	189.5 (152.7–249.5)	202.9 (157.4–251.1)
TEST (nmol/L)	1.64 (1.22–2.24)	2.72 (2.12–3.42)^*∗∗*^	2.97 (2.33–3.71)^*∗∗*^
LH (IU/L)	4.23 (3.02–6.09)	11.05 (6.40–14.00)^*∗∗*^	7.35 (4.67–11.60)^*∗∗*^^▲^
FSH (IU/L)	7.72 (6.73–9.14)	7.32 (6.03–8.87)	7.53 (6.33–8.97)
PRL (mIU/L)	372.9 (232.9–404.1)	290.2 (212.0–412.6)	412.3 (244.2–506.7)^*∗∗*^^∆^
FAI	2.56 (1.82–4.78)	6.37 (3.46–10.25)^*∗∗*^	9.28 (6.22–14.74)^*∗∗*^^▲^

BMI, body mass index; SBP, systolic blood pressure; DBP, diastolic blood pressure; WHR, waist hip ratio; FBG, fasting blood glucose; 2h-BG, 2-h post-glucose load blood glucose; FIns, fasting plasma insulin; 2h-Ins, 2 h plasma insulin after glucose overload; TG, triglyceride; TC, total cholesterol; HDL-C, high-density lipoprotein cholesterol; LDL-C, low-density lipoprotein cholesterol; FFA, free fatty acid; HOMA-_IR_, homeostasis model assessment of insulin resistance; AUC_glucose_, the area under the curve for glucose; AUC _insulin_, the area under the curve for insulin; VAI, visceral adiposity index; BAI, body adiposity index; SHBG, sex hormone-binding globulin; DHEA-S, dehydroepiandrosterone sulfate; TEST, testosterone; LH, luteinizing hormone; FSH, follicular stimulating hormone; PRL, prolactin; Prog, progesterone; FAI, free androgen index. Values were given as median (interquartile range). ^*∗*^*p* < 0.05;^*∗∗*^*p* < 0.01 compared with controls. ^*▲*^*p* < 0.05, ^*△*^*p* < 0.01 compared with *Q*1.

**Table 2:** Association of adipose IR with PCOS and *M* values in fully adjusted models.

Model adjust	*M* value		PCOS			
	OR	95% CI	*p*	OR	95% CI	*p*
BP	0.966	0.955–0.977	<0.01	1.038	1.022–1.053	<0.01
BP, BMI	0.975	0.963–0.987	<0.01	1.030	1.014–1.046	<0.01
BP, BMI, WHR	0.974	0.963–0.986	<0.01	1.031	1.015–1.048	<0.01
BP, BMI, WHR, SHBG	0.975	0.975–0.964	<0.01	1.032	1.014–1.049	<0.01
BP, BMI, WHR, SHBG, DHEA-S,	0.975	0.963–0.987	<0.01	1.032	1.014–1.05	<0.01
BP, BMI, WHR, SHBG, DHEA-S, TEST	0.975	0.963–0.987	<0.01	1.031	1.013–1.049	<0.01
BP, BMI, WHR, SHBG, DHEA-S, TEST, LH,	0.975	0.963–0.987	<0.01	1.041	1.00–1.063	<0.01
BP, BMI, WHR,SHBG, DHEA-S, TEST, LH, FSH	0.972	0.959–0.985	<0.01	1.047	1.023–1.072	<0.01
BP, BMI, WHR, SHBG, DHEA-S, TEST, LH, FSH, PRL	0.972	0.959–0.985	<0.01	1.046	1.022–1.072	<0.01
BP, BMI, WHR, SHBG, DHEA-S, TEST, LH, FSH, PRL	0.972	0.959–0.985	<0.01	1.048	1.022–1.075	<0.01
BP, BMI, WHR, SHBG, DHEA-S, TEST, LH, FSH, PRL, lipid profile	0.973	0.959–0.987	<0.01	1.046	1.018–1.074	<0.01

*M* values are defined as systemic IR.

**Table 3:** Clinical characteristics, laboratory parameters, and adipose IR levels before and after treatment with metformin in PCOS women.

Variable	Baseline	Posttreatment 3 months	Posttreatment 6 months
Betatrophin (*μ*g/L)	0.53 (0.41–0.62)	0.3 (0.2–0.42)^*∗∗*^	0.35 (0.28–0.38)^*∗∗*^
BMI (kg/m^2^)	24.0 (22.5–26.3)	23.0 (21.4–26.5)^*∗*^	22.4 (21.0–25.3)^*∗*^
SBP	115.0 (110.0–120.0)	114.5 (109.0–120.0)	118.0 (107.0–121.0)
DBP	75.5 (70.0–80.0)	78.5 (76.0–81.0)	78.0 (74.0–81.0)^*∗*^
WHR	0.86 (0.81–0.90)	0.87 (0.82–0.91)	0.85 (0.83–0.92)
*M* value (mg/kg/min)	4.9 (3.5–6.0)	5.6 (4.4–7.3)^*∗*^	6.3 (5.0–8.7)^*∗∗*^
FBG (mmol/L)	4.90 (4.47–5.44)	4.60 (4.29–4.81)	4.54 (4.33–4.89)^*∗*^
2h-BG (mmol/L)	7.50 (6.19–8.80)	—	6.90 (5.74–8.85)^*∗*^
FIns (mU/L)	16.1 (9.0–20.5)	12.7 (9.0–19.5)	10.9 (7.4–16.8)^*∗*^
2h-ins (mU/L)	126.5 (89.4–167.2)	—	112.2 (77.9–161.5)^*∗∗*^
SHBG (nmol/L)	32.1 (20.4–40.6)	40.0 (21.5–69.0)^*∗∗*^	59.4 (42.8–103.0)^*∗∗*^^∆^
DHEA-S (*μ*g/dl)	210.3 (157.4–264.7)	202.95(170.3–260.8)	191.25 (144.3–213.2)^▲^
TEST(noml/L)	2.78 (2.18–3.43)	2.74 (1.81–3.36)	2.52 (1.38–2.90)
LH (IU/l)	8.77 (4.79–12.66)	7.9 (4.58–10.10)	7.29 (5.22–8.50)^*∗*^
FSH (IU/l)	7.37 (6.63–8.97)	7.01 (6–8.39)	7.69 (5.78–8.34)
PRL(mIU/L)	314.18 (228.32–498.41)	320.01 (233.41–430.57)	323.19 (260.76–381.60)
FAI	9.19 (6.22–11.49)	5.53 (3.44–13.71)	3.89 (1.71–6.08)^*∗∗*^^∆^
TG (mmol/L)	1.54 (0.89–2.49)	0.89 (0.68–1.40)	0.96 (0.61–1.44)^*∗*^
TC (mmol/L)	4.46 (3.81–4.81)	3.99 (3.54–4.46)	3.88 (3.56–4.61)^*∗*^
HDL-C (mmol/L)	1.24 (1.07–1.38)	1.26 (1.1–1.52)	1.21 (1.12–1.49)
LDL-C (mmol/L)	2.40 (1.97–3.12)	2.17 (1.68–2.59)	2.11 (1.78–2.46)
FFA (*μ*mol/L)	0.62 (0.50–0.81)	0.52 (0.38–0.61)^*∗*^	0.54 (0.42–0.64)^*∗*^
HOMA-_IR_	3.61 (1.71–4.8)	2.65 (1.70–3.85)^*∗*^	2.31 (1.35–3.21)^*∗*^
AUC_glucose_	16.3 (14.1–19.0)	16.5 (15.0–18.7)	15.0 (13.7–16.9)^∆^
AUC_insulin_	207.3 (147.4–294.9)	189.1 (142.4–253.0)	171.3 (114.9–230.8)^*∗*^
VAI_female_	2.09 (1.35–3.31)	1.47 (0.88–2.22)	1.24 (1.01–1.81)^*∗*^
BAI	30.3 (27.3–32.9)	28.9 (25.6–31.8)	27.8 (26.1–30.4)^▲^
Adipose IR	64.4 (28.3–108.2)	37.8 (28.2–70.8)^*∗*^	41.0 (24.2–60.5)^*∗*^

^*∗*^ *p* < 0.05, ^*∗∗*^*p* < 0.01 compared with baseline; ^*▲*^*p* < 0.05, ^*△*^*p* < 0.01 compared with posttreatment 3 months.

## Data Availability

The data sets used and/or analyzed during the current study are available from the corresponding author upon reasonable request.

## Abbreviations

PCOS: Polycystic ovary syndrome
IR: Insulin resistance
Adipose IR: Adipose insulin resistance
FFA: Free fatty acid
BMI: Body mass index
PRL: Prolactin
TG: Triglyceride
HOMA-IR: Homeostasis model assessment of insulin resistance
AUC_glucose_: The area under the curve for glucose
AUC_insulin_: The area under the curve for insulin
VAI_female_: Visceral adiposity index
BAI: Body adiposity index
FAI: Free androgen index
SHBG: Sex hormone-binding globulin
LH: Luteinizing hormone.
